# New Antibody Conjugates in Cancer Therapy

**DOI:** 10.1100/tsw.2010.191

**Published:** 2010-10-12

**Authors:** Serengulam V. Govindan, David M. Goldenberg

**Affiliations:** ^1^ Immunomedics, Inc., Morris Plains, NJ, USA; ^2^ Garden State Cancer Center at the Center for Molecular Medicine and Immunology, Belleville, NJ, USA

**Keywords:** monoclonal antibody, conjugated antibody, radioimmunoconjugates, drug-conjugated antibodies, toxin conjugates, Dock-and-Lock technology, pretargeting, combination therapies

## Abstract

Targeting of radiation, drugs, and protein toxins to cancers selectively with monoclonal antibodies (MAbs) has been a topic of considerable interest and an area of continued development. Radioimmunotherapy (RAIT) of lymphoma using directly labeled MAbs is of current interest after approval of two radiolabeled anti-CD20 MAbs, as illustrated with the near 100% overall response rate obtained in a recent clinical trial using an investigational radiolabeled anti-CD22 MAb, ^90^Y-epratuzumab. The advantage of pretargeted RAIT over directly labeled MAbs is continuing to be validated in preclinical models of lymphoma and solid tumors. Importantly, the advantages of combining RAIT with radiation sensitizers, with immunotherapy, or a drug conjugate targeting a different antigen are being studied clinically and preclinically. The area of drug-conjugated antibodies is progressing with encouraging data published for the trastuzumab-DM1 conjugate in a phase I clinical trial in HER2-positive breast cancer. The Dock-and-Lock platform technology has contributed to the design and the evaluation of complex antibody-cytokine and antibody-toxin conjugates. This review describes the advances made in these areas, with illustrations taken from advances made in the authors' institutions.

Using monoclonal antibodies (MAbs) as carriers of tumoricidal entities is a topic of considerable current interest. With the U.S. Food and Drug Administration (FDA) approval of two radioimmunoconjugates, and with numerous therapeutic candidates at various stages of clinical testing, this area is ripe for additional regulatory approvals. This mini-review focuses on developments in antibody conjugates of radionuclides, drugs, and toxins, with emphasis on recent advances from the authors' work.

## RADIOIMMUNOCONJUGATES

Radioimmunotherapy (RAIT) involves the application of radiolabeled MAbs for targeted radiotherapy[1]. Both directly radiolabeled MAbs and *in vivo* radiolabelings of tumor-targeted MAbs by complexation with radiolabeled haptens ("pretargeting") have been developed.

## Radionuclides Used for RAIT

Continuous low-dose irradiation from a tumor-targeted radiolabeled MAb produces tumoricidal effects. For therapy, α- and β-particle emitters are of practical relevance. Table 1 shows some commonly used radionuclides. There have been numerous investigations with a number of these radionuclides, but for use with whole antibodies, the most promising and practical radionuclides are the β-emitters $^{131}$I, $^{90}$Y, and $^{177}$Lu. $^{90}$Y is a high-energy β-emitter (Emax: 2,280 keV; max range: 12 mm) with a 64-h half-life, while $^{131}$I has a higher half-life of 8.1 days with low-intermediate energy (610 keV, range: 2.0 mm). $^{131}$I is not suitable for use with internalizing MAbs because $^{131}$I is quickly removed from tumor cells after intracellular antibody catabolism. Intracellularly trapped forms of $^{131}$I have been designed by us and others for use with internalizing MAbs[2,3,4]. A recent study utilized the IMP-R4 template for incorporating residualizing radioiodine for immuno-PET quantitation of de2-7 EGFR expression in glioma in a xenograft model using $^{124}$I-IMP-R4–labeled anti-EGFR antibody, ch806[5]. The use of the residualizing radioiodine method for clinical RAIT may be supplanted by the availability of the metallic radionuclide $^{177}$Lu, which has radiophysical properties similar to those of $^{131}$I and radiolabeling chemistry similar to that of $^{90}$Y. Yet, the concept of residualizing radiohalogen is still of practical relevance with the α-emitting radiohalogen $^{211}$At. Vaidyanathan et al. demonstrated this using anti-EGFRvIII MAb, L8A4, radiolabeled with the residualizing $^{211}$At-SGMIB, in a xenograft model in nude mice[6].

**TABLE 1**
**Common α- and β-Emitting Radionuclides Used in RAIT Studies**

| Radionuclide | $t_{½}$ | Emission | $E_{max}$; $range_{max}$ |
|---|---|---|---|
| $^{131}$I | 8.0 days | β | 610 keV; 2.0 mm |
| $^{90}$Y | 64 h | β | 2,280 keV; 12.0 mm |
| $^{177}$Lu | 6.7 days | β | 496 keV; 1.5 mm |
| $^{225}$Ac | 10.0 days | α | 6,000–8,000 keV; 0.06–0.09 mm |
| $^{213}$Bi | 46 min | α | >6,000 kev; 84 μm |
| $^{211}$At | 7.2 h | α | 7,450 kev; 80 μm |

There have been numerous studies on α-particle RAIT. The advantage with using an α-emitter is that it deposits energy within a narrow path, leading to high linear energy transfer (high LET) within the target. In principle, this, coupled with short half-lives, can lead to less bystander toxicity to nontarget tissues than with MAbs labeled with β-emitters. $^{213}$Bi, $^{225}$Ac, and $^{211}$At are among the radionuclides most investigated in α-particle RAIT. Although $^{212}$Pb is a β-emitter and is used as an *in vivo* generator of $^{212}$Bi, which is an α-emitter, an interesting case is made for using $^{212}$Pb for RAIT[7]. Challenges abound in the use of α-particle RAIT. Availability of $^{211}$At is limited to centers with cyclotrons with 25–30 MeV α-particle beams. Radiolysis, stability issues, and chelate instability after decay to daughter radionuclides are other nontrivial issues. The promise and the challenge of using an α-particle emitter for RAIT have been described in many articles[7,8,9].

## RAIT of Hematological Cancers

$^{131}$I-Tositumomab (Bexxar®; GlaxoSmithKline) and $^{90}$Y-ibritumomab tiuxetan (Zevalin®; Spectrum Pharmaceuticals) are the two FDA-approved radiolabeled anti-CD20 murine antibodies that have shown significant overall and complete response rates in patients with non-Hodgkin lymphoma (NHL), a disease that is quite radiosensitive as well as readily accessible to the antibody conjugates[10]. Several hundred publications, including several reviews[11,12,13], can be gleaned in a PubMed search describing these agents and their use in combination with chemotherapy or as high-dose therapies in conjunction with autologous stem cell transplantation (ASCT).

A $^{90}$Y-labeled anti-CD22 MAb, epratuzumab ($^{90}$Y-epratuzumab tetraxetan), is in advanced clinical trials. Unlike $^{90}$Y-ibritumomab tiuxetan, where an acyclic MX-DTPA chelating agent is used, $^{90}$Y-epratuzumab tetraxetan contains the macrocyclic chelating agent DOTA[14]. A more stable attachment of radionuclide in $^{90}$Y-epratuzumab tetraxetan using the DOTA chelator has enabled higher doses to be administered safely in patients[15]. In addition, the idea of fractionated RAIT was instituted for a more uniform distribution of the dose within tumors[16]. Fractionated dosing also increased the administrable dose to 2.5-fold the single dose of this agent, which in itself was 20% higher than the approved single dose of $^{90}$Y-ibritumomab tiuxetan, and the increased dose entailed manageable toxicity. In addition, the antibody used is an internalizing humanized anti-CD22 MAb, hLL2 (epratuzumab), which does not generate neutralizing MAbs and improves the tumor residence time of the nuclide due to the residualizing nature of $^{90}$Y after internalization.

The combination of all these factors likely contributed to improved treatment outcomes documented in a recent clinical study. In this multicenter trial involving 64 patients with relapsed or refractory NHL, a 62% overall OR rate (48% CR/Cru rate) and a median progression-free-survival (PFS) of 9.5 months were recorded across all histological types with and without prior ASCT; in follicular lymphoma patients without prior ASCT, a 92% CR/CRu rate achieved with a cumulative dose of >30 mCi/m$^2$ was three times that reported with $^{90}$Y-ibritumomab tiuxetan[17]. The study concluded that 20 mCi/m$^2$ × 2 weeks was the recommended fractionated dose for future studies.

## Pretargeting

Pretargeted RAIT is a technique that allows the antibody localization phase to be temporally separated from the radionuclide administration in the form of a small molecular hapten. A number of specialized reports describe different formats of pretargeting, notably the bispecific antibody (bsAb) approach from our laboratories and the streptavidin (sAv)-biotin based method from others[18,19,20].

## Dock-and-Lock Technology to Prepare Fusion Proteins for Targeted Therapies

A new technology, termed “Dock-and-Lock” (DNL), has been devised, which involves a facile and flexible method of assembling multifunctional structures based on noncovalent interaction between a regulatory subunit of cAMP-dependent protein kinase A (dimerization and docking domain, “DDD”) and the anchoring domain, “AD”, of A kinase anchor proteins, AKAPs[21,22]. By adding one functional moiety to the AD domain and a second functional moiety to DDD, the latter always being in dimeric form and thus displaying two copies of the second functional moiety, complex multifunctional structures are readily assembled. The noncovalent interaction between the AD module and the dimeric DDD2 module (“docking”) is further stabilized by two disulfide bonds (“locking”) formed between two sets of cysteine groups engineered onto each of these modules. Whole antibody and/or antibody fragment can be readily incorporated in the assembly. Fig. 1 illustrates the DNL technology using the TF2 fusion protein. The application of DNL technology to rapidly assemble fusion protein conjugates for targeted therapies is described below and in subsequent sections.

## Application of DNL to Pretargeted RAIT

The DNL technology provides a facile access to tri-Fab structures for pretargeted RAIT by the bispecific antibody (bsAb) approach. For preclinical therapy of lymphoma, a tri-Fab, TF4, was employed. TF4 is a

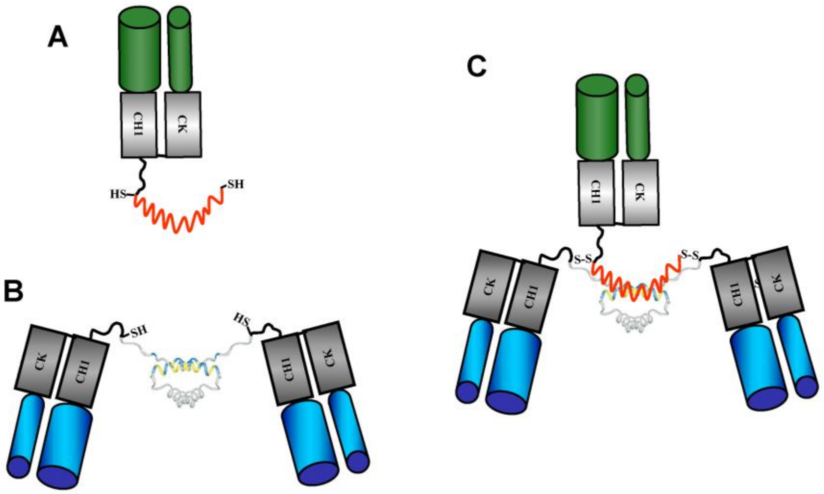

**FIGURE 1.** Representations of h679-AD2 (A), C-DDD2-hMN-14 (B), and the bispecific tri-Fab fusion protein, TF2 (C), prepared by the DNL method. In this, one Fab fragment, derived from humanized 679 MAb with binding specificity for HSG, is attached to the AD2 peptide. Two Fabs are derived from humanized MN-14 MAb, with binding specificity for CEACAM5, and are on the DDD dimer. Noncovalent binding between the functionalized DDD2 and AD modules and the subsequent locking disulfide formations lead to the stable TF2 fusion protein. Reproduced from Figure 2, Rossi et al.[22], with permission. Copyright 2006 by the National Academy of Sciences, U.S.A.

fusion protein with two Fabs specific for the CD20 antigen, while the third Fab is specific for a hapten incorporated in a peptide, and the latter, used as the second step agent in pretargeting, is designed for a fast binding to the tumor-localized tri-Fab bispecific and a fast clearance from blood to result in high tumor-nontumor uptake ratios. The hapten is histamine-succinyl-glycine (HSG) and the peptide IMP-288 carries two HSG moieties as well as a DOTA moiety for chelation to therapeutic radionuclides, such as $^{90}$Y. After a localization and clearing phase, radiolabeled IMP-288 is administered after the serum level of the pretargeted bsAB is at or below 1%. The bis-HSG–containing peptide (the bivalent hapten) binds two molecules of the tumor-targeted bsAb, such as TF4, in what is referred to as the Affinity Enhancement System ("AES"[23]). The bsAb method of pretargeting has been well described[24,25]. The advantages of the bsAb approach are that it does not require a clearing agent and does not use streptavidin of the sAv-biotin approach, where immunogenicity due to sAv is a problem[26]. Fig. 2 gives a cartoon illustration of pretargeting by the bsAb approach.

## Pretargeted RAIT of NHL

NHL therapy has been examined in detail by pretargeting. In a conventional NHL model in nude mice, pretargeting with TF4, followed by $^{90}$Y-IMP-288, led to dramatic cure rates compared to therapy with direct $^{90}$Y-veltuzumab, which is the radiolabeled anti-CD20 antibody hA20 (veltuzumab[27]). Similar high cure rates were also reported using the sAv-biotin format of pretargeting in the Ramos lymphoma model, using the anti-CD20 antibody-sAv fusion protein 1F5(ScFv)$_4$SA and the $^{90}$Y-DOTA-biotin pair[28]. In a more recent study, Pagel et al. compared direct vs. pretargeted RAIT in xenograft models of lymphoma using CD20, CD22, and HLA-DR as the targets, and showed that pretargeted RAIT by the sAv-biotin approach produced higher therapeutic indices and superior tumor regression, and PFS vs. direct RAIT for the CD20 or HLA-DR targeting[29]. These authors make a clear case for pretargeted RAIT for clinical therapy of NHL. In an editorial on this article, it was observed that dose fractionation and combination therapies should further enhance the advantage of pretargeted RAIT[30].

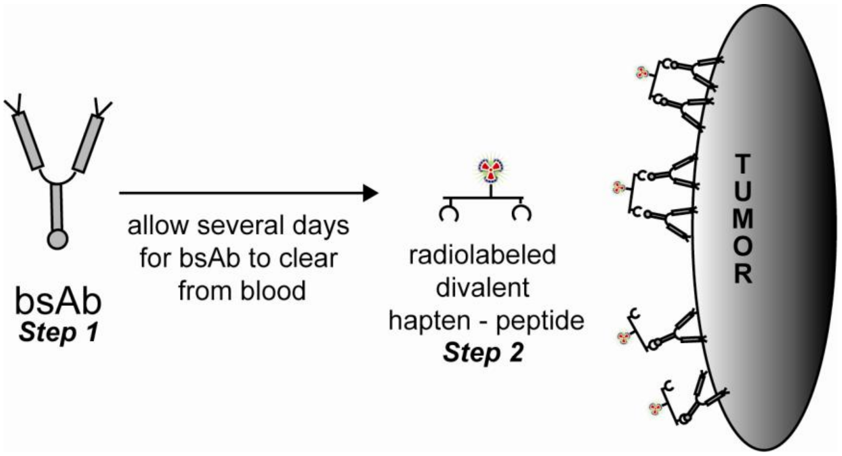

**FIGURE 2.** In pretargeting by the bsAb approach, a bsAb is first injected. After tumor targeting and clearance of the bsAb from circulation, a peptide with two hapten moieties and a therapeutic or a diagnostic radionuclide is administered. One bivalent hapten can cross-link two closely spaced bsAb molecules on the tumor cell surface.

## Miscellaneous Aspects

Hematological cancers other than NHL also have been treated by the RAIT modality. Despite very promising clinical outcomes, the approved radioimmunoconjugate products have not been well received in the marketplace for reasons that are not related to product performance. The slow acceptance of these products may be due, in part, to their intended use for NHL, for which conventional chemotherapy, alone or in combination with the approved anti-CD20 immunotherapy rituximab, has led to long durations of disease control. It has been remarked that perseverance with continued documentation of the superior safety and efficacy profiles obtainable with RAIT, particularly pretargeted RAIT, together with defined roles for oncologists as well as nuclear medicine specialists in the disease management, should lead to a more enthusiastic acceptance of this treatment modality[30].

## RAIT of Solid Cancers

RAIT as a therapy modality could be useful in the treatment of nonhematological malignancies as well, but as yet has had less convincing results. A clinical trial using $^{90}$Y-humanized PAM4 ($^{90}$Y-clivatuzumab) in fractionated doses in combination with the radiosensitizer gemcitabine is ongoing. The rationale for fractionated dosing combined with gemcitabine stemmed from convincing preclinical data[31,32]. In a phase Ib trial, 27 naïve patients with advanced metastatic pancreatic cancer (stage III or IV) were treated with 6.5, 9.0, 12.0, or 15.0 mCi/m$^2$ of $^{90}$Y-clivatuzumab tetraxetan ($^{90}$Y-hPAM4) every week on weeks 2–4, in combination with 200 mg/m$^2$ of gemcitabine given once weekly for 4 weeks[33]. Overall, 68% of the 22 evaluable patients showed either tumor shrinkage or disease stabilization, with one-third of these clinical benefits being partial responses as per RECIST criteria. Treatment with higher doses gave better outcomes than with lower doses. Treatment was well tolerated in spite of higher cumulative $^{90}$Y doses, with manageable and reversible thrombocytopenia or neutropenia. Anecdotal reports, which need to be validated, indicated reduced pain medication during treatment. These results are promising in a disease indication where life expectancy is 6–12 months from diagnosis. Thus, RAIT may prove useful in improving quality of life indices and extending survival.

Karacay et al. reported recently on the preclinical therapy of a pancreatic tumor model using the tri-Fab TF10 for pretargeting, followed by $^{90}$Y-IMP-288[34]. TF10 has bivalent specificity for mucin antigen on pancreatic tumors and is based on PAM4 antibody and monovalent specificity for HSG. In the Capan-

1 human pancreatic cancer model in nude mice, pretargeted RAIT was compared with directly labeled $^{90}$Y-hPAM4 for efficacy and toxicity using a sub-MTD with pretargeted RAIT and a dose of directly labeled hPAM4 that was at or above MTD. Eight of ten animals treated by pretargeted RAIT and eight of nine animals treated with $^{90}$Y-hPAM4 were tumor-free after 30 weeks, but the hematological toxicity was more pronounced and the recovery phase was longer in direct vs. pretargeted therapies. No evidence was found of renal toxicity, a usual concern with pretargeted RAIT. The authors concluded that pretargeted RAIT had the promise of equivalent efficacy to direct RAIT, but with less systemic toxicity. Combination with gemcitabine and fractionated pretargeted RAIT contributed to enhanced responses.

## Methods to Enhance Solid Tumor Therapy

It has been suggested that combination therapies, pretargeting strategies, locoregional applications, α-particle RAIT, and application to minimal residual disease would prove to be valuable[35]. The promise of RAIT in an adjuvant setting[36,37] and other methods for improving efficacy in solid cancers have been described in a previous review[38].

## Potential of DNL in Pretargeted Diagnostic Imaging

This topic is of much importance as a means of not only diagnosing sites of cancer in patients, but also for using it as a guide to determine therapy outcomes. Applications of the tri-Fab structures for imaging purposes using SPECT and PET radionuclides have been described[39,40]. The first results from pretargeted SPECT imaging in colorectal cancer patients using the DNL approach to pretargeting have been reported[41]. In this, 75 mg of TF2 (a tri-Fab with two arms for binding to CEACAM5 and one arm for binding to HSG) were used for pretargeting, followed 5 days later by the administration of 5 mCi of $^{111}$In-IMP-288. All known sites of disease were targeted. This first clinical validation of DNL technology confirmed the safety and specific targeting in the pretargeting format. A comprehensive review on DNL in pretargeted RAIT has also appeared[42]. A significant new approach to using $^{18}$F for PET imaging has been developed, using a rapid and convenient method for labeling small molecules with $^{18}$F for pretargeted PET imaging of cancers by the bsAb approach described earlier. The salient features of this method, which is also applicable to receptor targeted peptides, are described below.

## A Novel $^{18}$F PET Imaging by Pretargeting for Use with Therapy

In cancer therapy, treatment effectiveness is often determined by computed tomography and more recently by $^{18}$F-FDG PET imaging. Although FDG is useful in determining the sites of glucose uptake, with the tumor representing high glucose utilization, its application is nonspecific, since FDG is also taken up in inflammatory sites. A pretargeting approach, in combination with a $^{18}$F-labeled second step agent, could be of value by combining antibody specificity with the sensitivity of PET. A significant new advance in this regard has been made by McBride et al. in the $^{18}$F labeling of small molecules, which replaces the multiple synthetic and purification steps of current methods with a rapid and facile labeling[43]. The method involves chelation of Al-$^{18}$F complex to a NOTA-derivatized peptide. The method was illustrated with $^{18}$F labeling of a bis-HSG-peptide, IMP-449, incorporating a NOTA derivative, and the labeled peptide was used in a pretargeting format to target the LS174T human colon carcinoma xenograft in nude mice. Excellent tumor-nontumor ratios achieved with pretargeting, together with the sensitivity of $^{18}$F PET, led to exceptional PET imaging of the tumor with virtually no background activity; this was contrasted with the metabolic imaging with $^{18}$F-FDG PET that gave considerable uptake in bone marrow, heart, and brain, as well as in tumor. Tumor/blood ratios for pretargeting and FDG PET were 249:1 and 10:1, respectively, when the animals were imaged 2 h after the administration of radioactivity. The article demonstrated the easy labeling method, which provided high specific activity of the labeled peptide in a short duration. Application to other pretargeting systems as well as receptor-targeted peptides are forthcoming[43]. More refinement of this labeling process was also reported recently, with radiolabeling accomplished within 30 min[44]. The process achieved a high specific activity and did not need HPLC purification.

## ADCs (ANTIBODY-DRUG CONJUGATES)

Using antibodies to carry highly toxic drugs has gained interest recently. The impetus for this activity stemmed from the realization that highly toxic drugs would be needed for targeted therapy using MAbs as carriers[45]. This is because of low levels of localization of antibodies at the tumor sites, which would be compensated by high toxicity of the drug. Usually, these drugs are not useable as such in view of a poor therapeutic index. The structures of some ADCs are given in Fig. 3.

## Conjugates of DNA Minor Groove Binder, Calicheamicin

Gemtuzumab ozogamicin (Mylotarg®; Pfizer) was the first FDA-approved drug immunoconjugate targeting CD33 and carrying the highly toxic calicheamicin, which is a DNA minor groove binder[46]. This conjugate was granted accelerated approval, subject to postapproval studies to confirm the product's clinical benefit. The drug has picomolar $IC_{50}$ and it is attached to the humanized anti-CD33 MAb hP67.6, with cleavable hydrazone and disulfide bonds. In CD33-positive acute myeloid leukemia (AML) patients over the age of 60 and in relapse, a phase II clinical trial showed a median 6.8 months relapse-free survival and a 30% remission rate. Considerable data have been accumulated postapproval, defining the scope and limitations of this agent in AML[47].

It was previously determined that gemtuzumab ozogamicin was clinically effective in leukemia blasts that lacked CD33 expression, which was also subsequently demonstrated in preclinical xenograft models that were negative for CD33 expression[48]. In this article, Boghaert et al. showed that the lack of specificity was confined only to IgG conjugates of calicheamicin, and that an HSA conjugate or a conjugate of pegylated Fc fragment were not effective. They concluded that the high potency of the drug, together with the acid-cleavable nature of the construct and the IgG form, contributed to the conjugate's nonspecific efficacy by an enhanced permeation and retention effect.

Recently, market withdrawal of Mylotarg® was announced because postapproval studies (SWOG S0106) did not demonstrate a survival advantage for the combination of immunoconjugate therapy and chemotherapy vs. chemotherapy alone, while the fatal toxicity rate was higher in the combination therapy arm.

A calicheamicin derivative with the same linker chemistry is also being examined clinically as an anti-CD22 conjugate, CMC-544, for the treatment of NHL. The efficacy of this conjugate was previously demonstrated in preclinical models of lymphoma either as a monotreatment or in combination with rituximab[49,50]. Results from a clinical phase I study of this agent, inotuzumab ozogamicin, have been published recently[51]. In this 79-patient study, the MTD was 1.8 $mg/m^2$, given every 3 or 4 weeks, with thrombocytopenia, asthenia, nausea, and neutropenia being the common adverse events. The majority of patients (80%), treated every 3 weeks, experienced grade 3 or 4 adverse events, which was attributed to nontarget effects of the conjugate. The overall response rates and CR/CRu rates in a follicular lymphoma patient subset (n = 22) receiving the MTD were 68 and 32%, respectively, and in a diffuse large B-cell lymphoma (DLBCL) patient subset (n = 26) treated at the MTD, these response rates were 15 and 7.7%, respectively. Median PFS in the follicular (n = 18) and the DLBCL (n = 25) patient subsets were 317 and 49 days, respectively.

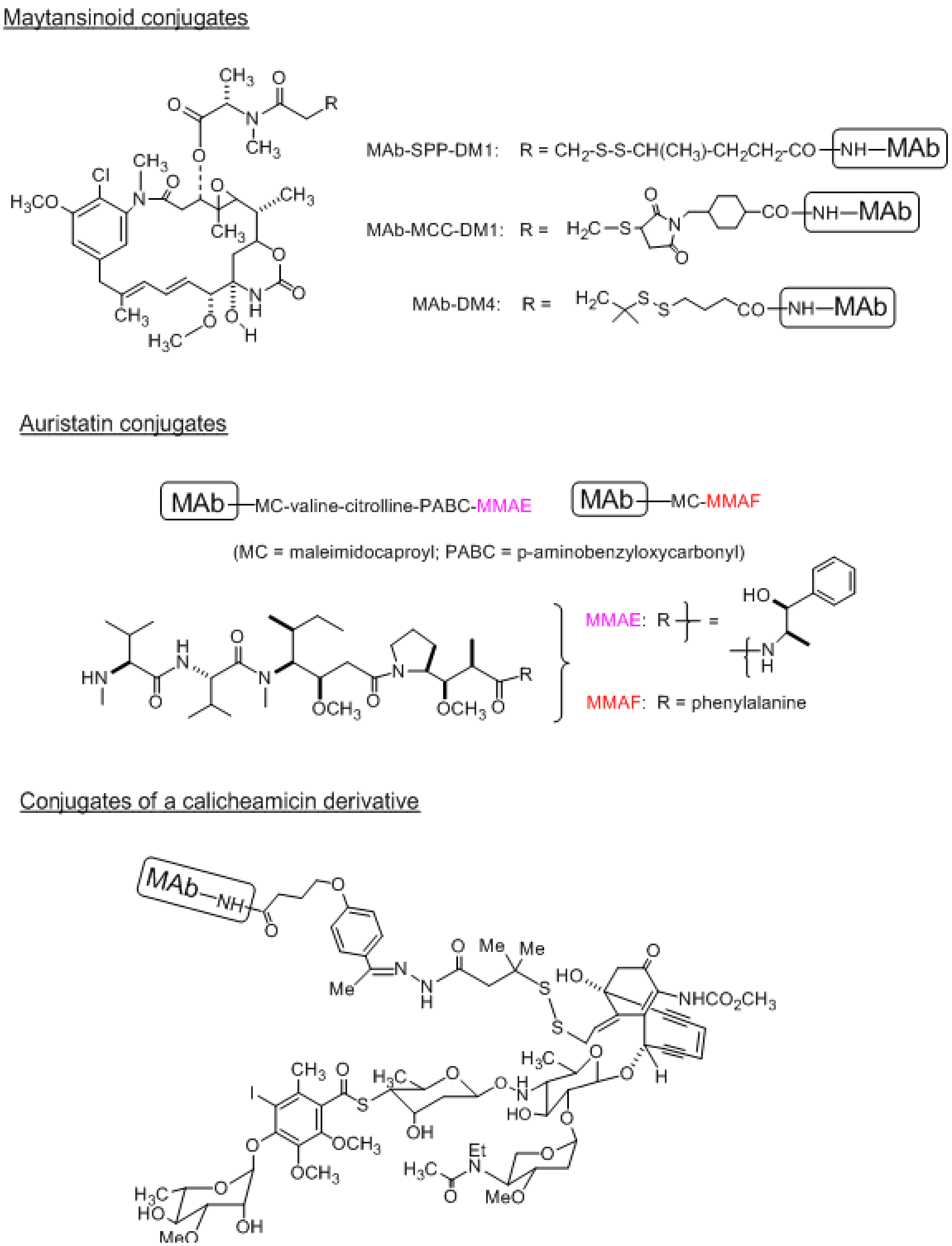

**FIGURE 3.** Antibody conjugates incorporating highly potent maytansinoids (top), auristatins (middle), or a calicheamicin derivative (bottom).

When compared to the clinical result with this drug conjugate, the outcome in the fractionated RAIT using $^{90}$Y-epratuzumab in NHL patients (described earlier[17]) is quite favorable: 62% overall OR rate

(48% CR/Cru rate) and a median PFS of 285 days for patients across all histological types of NHL with and without prior ASCT, and a 92% CR/CRu rate in follicular lymphoma patients without prior ASCT, with a median PFS of 738 days, with manageable toxicities.

## Moderately Cytotoxic Drug as a Conjugate of a Unique Carrier MAb

Calicheamicin and other toxic drugs currently explored by other groups have picomolar or subnanomolar $IC_{50}$s. Yet, in some situations, moderately toxic drugs, such as doxorubicin, that are used for systemic chemotherapies could work well if a high tumor concentration of the drug can be achieved. We indeed found this to be the case preclinically when using a unique anti-CD74 antibody, hLL1 (milatuzumab), as a carrier of doxorubicin[52,53]. This antibody is unique among internalizing antibodies in that $10^7$ molecules of antibody per cell per day are internalized, routed to lysosomes, and degraded[52]. The exceptional therapeutic efficacies obtained by targeting CD74 by the anti–CD74-doxorubicin conjugate hLL1-doxorubicin in preclinical models of lymphoma[54] and multiple myeloma (MM)[55] were described previously[38]. Naked hLL1 is now being investigated clinically in MM and chronic lymphocytic leukemia (CLL) patients, and early results show indication of disease stabilization in MM at 4 and 8 mg/kg doses given twice weekly for 4 weeks[56]. The antibody's promise is in its capacity to localize therapeutic concentrations of the drug in tumors. A phase I/II study of hLL1-doxorubicin conjugate for MM therapy has begun (www.clinicaltrials.gov identifier: NCT01101594).

## Antibody Conjugates of Topoisomerase I Inhibitor SN-38

For solid tumor therapy, we have embarked on a program to evaluate SN-38 conjugates of our proprietary antibodies targeting antigens in solid tumors. SN-38 is the active drug form of irinotecan (CPT-11), a prodrug used in systemic chemotherapy of metastatic colon cancer. The active drug, SN-38, is two to three orders of magnitude more potent than the prodrug with single-digit nanomolar $IC_{50}$ in various cell lines *in vitro*. SN-38 belongs to the camptothecin group of alkaloids that exhibit antitumor effects by inhibiting the recoiling of the DNA-topoisomerase I complex during replication, thereby producing double strand breaks. There are numerous studies documenting the fate of CPT-11 and SN-38 in liver and intestines[57,58].

The design of a series of bifunctional SN-38 derivatives, with cross-linker attachment at the drug's 20 position, and their MAb conjugates, was reported[59]. Subsequently, SN-38 conjugates with antibody attachment at the 10 position of the drug were also prepared. Structures of SN-38 conjugates examined by us are given in Fig. 4. Conjugates attached at the 20 position of the drug, either in the form of an ester or a carbonate, showed antitumor activity, while those with attachment at the 10 position in the form of carbamates were inactive[60]. One of the linkers in the latter category, "CL2D", was inert to the action of cathepsin-B, corroborating a similar finding in another system[61]. However, the lack of potency with a linker, "CL2E", that was readily cleaved by cathepsin-B and that had a dimethylethylenediamine spacer for internal cyclization to liberate SN-38 intact was unexpected. Whether the inefficiency in intact liberation of the drug is unique to MAb-SN-38 conjugates is unclear.

In the case of SN-38, the best linker was CL2. In xenograft models, we have shown significant and selective therapeutic efficacies using SN-38 conjugates of MAbs. In a lung metastatic murine model of human colon carcinoma, the median survival time (MST) for untreated animals was 45 days, that of specific conjugate was 145 days, while nonspecific conjugate showed MST of 73 days[62]. Kaplan-Meier survival curves due to different treatments in this experiment are shown in Fig. 5. The cross-linker in these conjugates was further optimized to "CL2A", which was a phenylalanine-deleted variant of the CL2 linker, and the conjugates with either the CL2 or CL2A linker showed equivalent efficacies preclinically *in vitro* and *in vivo*[63]. Encouraging efficacy and an excellent therapeutic window documented in preclinical therapy studies[63] auger well for examining these conjugates clinically.

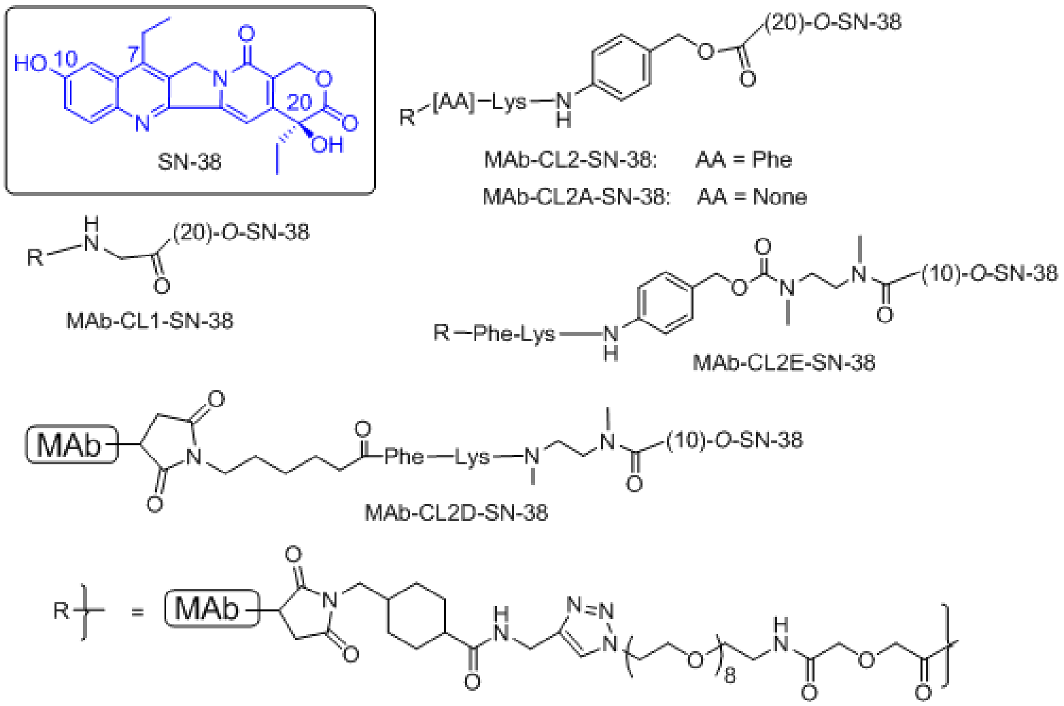

**FIGURE 4.** Antibody conjugates of some SN-38 derivatives.

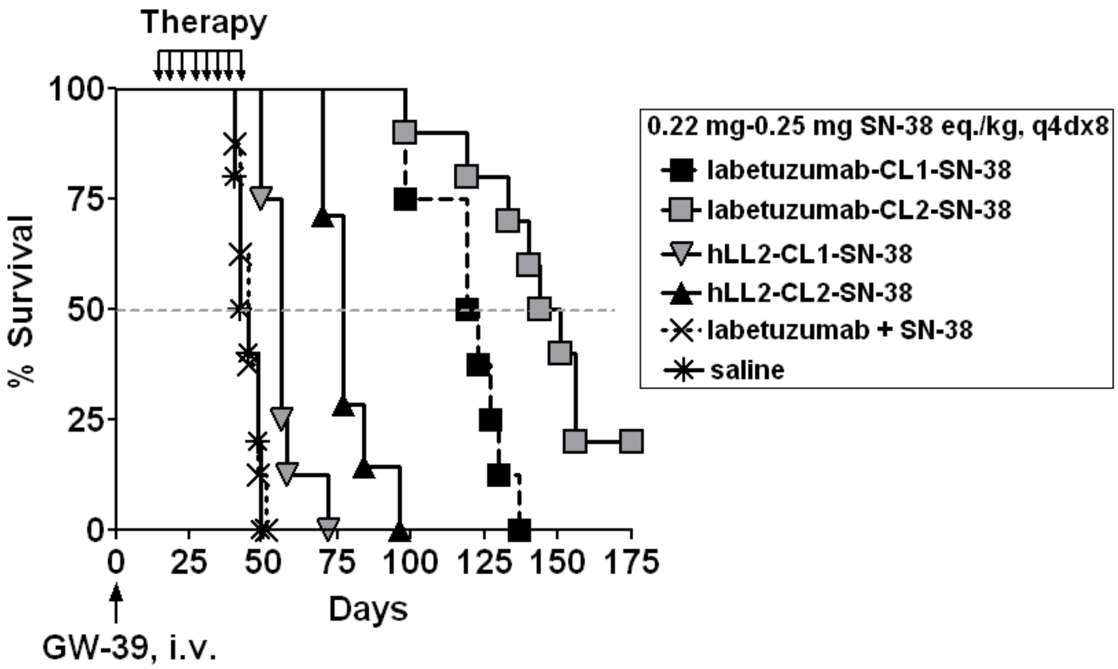

**FIGURE 5.** In a lung metastatic model of the GW-39 human colon carcinoma in nude mice (n = 7–10), animals were administered CL1-SN-38 or CL2-SN-38 conjugates of specific labetuzumab (hMN-14), respective nonspecific conjugates of epratuzumab (hLL2), and other controls as noted. The dose and dose schedule were 0.22–0.25 mg SN-38 equivalent/kg, twice weekly for 4 weeks. The figure shows Kaplan-Meier survival plots for different treatments. Reproduced from Figure 2B of Govindan et al.[62].

## Using the Conjugate of a Prodrug of a DNA Alkylating Agent

Another approach to ADCs using supertoxic drugs has been one of masking the toxicity of the drug in the form of a prodrug. A DNA minor groove binding alkylating cytotoxic drug, structurally related to duocarmycin, was protected in the form of an aromatic carbamate, requiring the cleavage by a carboxylesterase, and this protected drug is conjugated to MAbs via an enzyme-cleavable linker. In preclinical models, this approach showed excellent therapeutic effects[64,65]. In patients, the tumor levels of human carboxylesterase necessary to convert the prodrug to the active drug can be expected to be a factor determining efficacy. Because of the protection in the form of a prodrug, the conjugates prepared by this approach have been shown to be well tolerated[65].

## Maytansinoids

Maytansinoids are highly potent drugs that act by inhibiting tubulin polymerization. Antibody conjugates using maytansinoids have been designed with three distinct linkers, the structures of which are given in Fig. 3. One of the linkers has a relatively labile disulfide bond, another contains a hindered disulfide bond, and yet another has a stable thioether bond[45]. The drug linker, liberated after lysosomal degradation, contains a lysine adduct of the linker drug. In the case of DM1 or DM4, further reduction by intracellular thiols, such as glutathione, liberated membrane-permeable DM1 or DM4, which sterilized even tumor cells that did not express the target antigen. Intracellular processing was demonstrated[66,67].

In a preclinical HER-positive breast cancer model, trastuzumab-maytansinoid conjugates with different cross-linkers were evaluated. It was found that the conjugate with the stable, noncleavable, thioether linker, “MCC-DM1”, was more efficacious and better tolerated than the conjugates with the cleavable disulfide linkers[68]. This is the basis for clinically evaluating the trastuzumab-maytansinoid conjugate, T-DM1, incorporating “MCC-DM1”. In a phase I clinical study of T-DM1 in metastatic breast cancer patients (n = 24) who had previously undergone traztuzumab-based therapies, immunoconjugate dosings every 3 weeks in the range of 0.3 to 4.8 mg/kg protein dose of the conjugate were examined[69]. The MTD was determined to be 3.6 mg/kg. Six out of 24 patients had objective partial responses (25%), and the combined partial response and stable disease rate was 73% among the cohort receiving the MTD. Thrombocytopenia was stated to be a key dose-limiting toxicity and reversible elevation of hepatic transaminases also was found.

## Auristatins

This is another class of supertoxic drugs, acting by inhibiting tubular polymerization, which is being extensively examined in the ADC format[70]. Conjugates of various auristatin derivatives have been evaluated, with those of two derivatives, MMAE and MMAF, being particularly noteworthy[71,72,73]. MMAF differs from MMAE by possessing a phenylalanine at the C-terminus, contributing to membrane impermeability. Interestingly, MMAF could be derivatized at the amine terminus with a noncleavable linker without loss of drug activity, which was not the case with the MMAE analog. The ADC of the latter would need to contain a cleavable linker. A salient feature in these investigations has been the documentation of higher toxicity with higher drug substitution, and lower toxicity and equivalent efficacy with lower substitution[70,74]. There have been a number of approaches taken to optimize the drug substitution level and an interesting new development pertains to the design of “thiomabs”, with two engineered cysteine groups on the variable region of MAb, leading to site-specific attachment of drugs, exemplified with auristatins[75,76].

The ADC SGN-35, composed of the chimeric anti-CD30 MAb cAC10 and the auristatin derivative MMAE, has produced promising clinical results in CD30-positive cancers. In a clinical phase I study in patients with relapsed or refractory Hodgkin lymphoma or systemic anaplastic large cell lymphoma, dose escalation in the 0.4 mg to 1.0 mg/kg range was examined in a once-weekly dosing schedule[77]. In this 17-patient study, the treatment was well tolerated and seven of eight evaluable patients in the two high-dose groups achieved CRs.

## Cleavable vs. Noncleavable Linker in Conjugates

Conjugates with a noncleavable linker may have an advantage of less toxicity vs. the conjugates with a cleavable linker, since these have to be specifically internalized for detachment from the carrier antibody, while the amino acid–appended metabolite of the drug, generated after intracellular metabolism, would be unable to diffuse through cell membranes. The greater efficacy and tolerability of trastuzumab-MCC-DM1 with a stable linker vs. maytansinoid conjugates with cleavable disulfide linkers was noted in a previous section[68]. Polson et al. compared the conjugates of both maytansinoids as well as an auristatin group of tubulin polymerization–inhibiting drugs, using cleavable and noncleavable linkers in NHL models[78]. Conjugates of maytansinoids with a stable linker (MAb-MCC-DM1) or a cleavable disulfide bond (MAb-SPP-DM1) were evaluated. Likewise, in the auristatin group, MMAE conjugates contained a cleavable linker, while MMAF conjugates contained a stable linker. It was found that anti-CD22 conjugates of these drugs with noncleavable linkers were less toxic in rats than those with cleavable linkers with both groups of drugs, when administered at single i.v. dose of 20 mg protein dose per kilogram. The study also found that ADCs with cleavable linkers were effective in a broad range of targets, while those with noncleavable linkers were more selective in the tumor targets that could be treated. Thus, which type of linker to use is a function of the specific tumor target, the tolerability of drug to substitution, and the therapeutic window achievable.

## ANTIBODY-TOXIN CONJUGATES

Different classes of toxins have been conjugated to MAbs[79,80,81]. Plant toxins, such as ricin, prevent the attachment of elongation factors 1 and 2 to a ribosome subunit and induce apoptosis; bacterial toxins, exemplified by *Pseudomonas* exotoxin (PE), inhibit protein synthesis; and ribonucleases, RNAse, promote caspase activation and apoptosis by damaging tRNA. The plant toxins contain both binding and catalytic domains. Binding domain–deleted versions are incorporated as fusion proteins or as chemical conjugates of antibodies or antibody fragments. Issues pertaining to toxin conjugates are antibody internalization, need for the catalytic domain to translocate to cytosol after antibody internalization, nonspecific toxicity, and methods to minimize these.

There is one approved toxin conjugate, denileukin diftitox, which is a fusion protein of human interleukin-2 and truncated diphtheria toxin, with efficacies observed in the treatment of cutaneous T-cell lymphoma, NHL, and CLL.

A recombinant anti-CD22 immunotoxin of PE38, BL22, has been evaluated in a phase II study in chemoresistant hairy cell leukemia patients[82]. In this clinical trial, patients (n = 36) were administered 40 μg/kg every 2 days for three doses in one cycle, with some patients receiving additional cycles of therapy. A CR rate of 25% and a PR rate of 25% were found after one cycle of therapy, which improved to a CR rate of 47% and a PR rate of 25% when 56% of patients were retreated. Best results of a 95% OR rate and a 64% CR rate were found in a subset of patients whose baseline spleen size was normal or only moderately enlarged.

## Ribonuclease Conjugates and Immunotoxin Design using DNL

Ranpirnase (rap) is a member of the pancreatic RNase superfamily. A fusion protein of rap (frog RNase) and milatuzumab, hLL1, was previously shown to be highly potent in a lymphoma model *in vivo*[83].

This fusion protein contained two rap molecules on the light chains of the IgG4 form of the antibody. More recently, MAb-rap fusion proteins were designed by the DNL approach. In these, an AD2 module is attached to each of the Fc regions of the antibody and when fused with rap-DDD2, the resultant construct displays a targeting IgG with four rap molecules fused to it. In this way, rap fusion proteins of anti-CD22 MAb, hLL2 (epratuzumab), anti-CD-20 MAb, hA20 (veltuzumab), anti-HLA-DR MAb, hL243 (IMMU-114), anti-CD74 MAb, hLL1 (milatuzumab), anti-EGP-1 MAb, hRS7, and anti-CEACAM5 MAb, hMN-14 (labetuzumab), were designed[84], and were designated 22-rap, 20-rap, C2-rap, 74-rap, 14-rap, and E1-rap, respectively. In lymphoma cell lines, 100% cell killing, *in vitro*, was established at concentrations ≥1 nM of the specific constructs. Using E1-rap, specific for EGP-1 (TROP-2) antigen, subnanomolar potencies ($EC_{50}$) were documented in various cell lines, which were three to four orders of magnitude more potent than free rap[85]. These promising *in vitro* data with these unique constructs suggest a need for more extensive preclinical evaluation.

## TARGETED CYTOKINE THERAPY USING DNL

The cytokine interferon-α (IFN-α) is known to exhibit antitumor effects in xenograft models[86] and in patients[87]. To overcome its short circulation half-life and to enhance efficacy, IFN-α has been conjugated to macromolecules, such as antibodies. MAb conjugates of IFN-α, constructed by chemical[88,89] or recombinant methods[90], have been evaluated preclinically.

An IgG-$(\text{IFN-}\alpha\text{-2b})_4$ fusion protein has been designed by attaching AD modules on the Fc regions of antibodies and fusing them by DNL with a dimeric DDD module on each AD, resulting in four moieties of cytokine per IgG molecule. Rossi et al. showed that the anti-CD20 MAb/IFN fusion protein, "20-2b", was significantly more potent in three lymphoma xenograft models than equidoses of the parent MAb, the cytokine, or an irrelevant IgG-IFN conjugate[91]. For example, in the early disseminated Daudi lymphoma model in SCID mice, with 0.7 pmol of the fusion protein given as a single s.c. dose, median survival was not reached on day 133 (7/10 animals alive), while median survival in animals given saline (untreated) or the equivalent dose of the anti-CD20 antibody, hA20, or nontargeting fusion protein, 734-2b, was 27–34 days. The power of targeting IFN for therapy was also established in a more advanced disease model, whereby the specific 20-2b treatment led to 100% survival on day 105, while treatment with an equidose of IFN using PEGASYS, a currently marketed pegylated IFN, was ineffective and the MST was only 42 days. Moreover, the same selective therapeutic efficacy was also demonstrated in two other models that were insensitive to IFN treatment, with one of the tumor models additionally refractive to anti-CD20 therapy.

## COMBINATION THERAPIES

Combination regimens have been a mainstay of cancer chemotherapy. In a similar fashion, it should be expected that combination therapies would be beneficial in targeted therapies as well. As drugs can act as radiosensitizers, this strategy formed the basis for examining the combination of RAIT with chemotherapy[92,93]. More recent examples of combination therapies are given below.

### Combination of Lymphoma RAIT with Immunotherapy

An interesting preclinical study combining $^{90}$Y-epratuzumab tetraxetan RAIT with anti-CD20 immunotherapy found a remarkable 80% cure rate (n = 15), lasting 100 days, in the aggressive Ramos NHL xenograft model[94,95]. The rationale for this combination is that RAIT and immunotherapy targeted different antigens; while $^{90}$Y with its long path length is suitable for reducing large tumor burden based on targeting CD22, single cells should be amenable to immunotherapy with a humanized anti-

CD20 MAb, veltuzumab. Moreover, the antibodies have different mechanisms of action. In the combination, the animals were administered 1, 0.5, 0.5, and 0.5 mg of veltuzumab on days 0, 7, 14, and 21, respectively, and with a single 175 µCi dose of $^{90}$Y-epratuzumab tetraxetan ($^{90}$Y-DOTA-hLL2) on day 1. Twelve of 15 mice were tumor free at the end of the experiment on day 168, while 12/15 untreated animals had to be humanely sacrificed by day 20 due to tumor growth. Fig. 6 shows the effect of combination therapy. Based on these promising preclinical data, a clinical study using this combination therapy has started (www.clinicaltrials.gov identifier: NCT01101581).

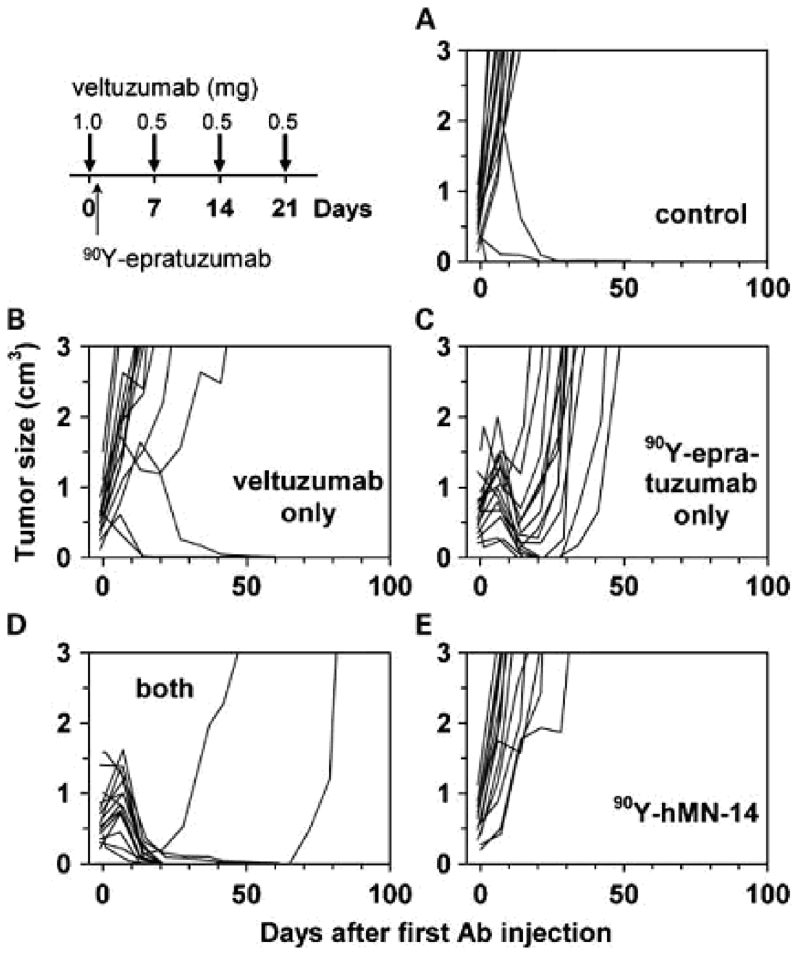

**FIGURE 6.** Therapy of s.c. Ramos tumors in nude mice (n = 13–15) with a combination of $^{90}$Y-epratuzumab (0.175 mCi) and the indicated doses of unconjugated veltuzumab (hA20). Sequence of administrations is indicated top left. Panels are self-explanatory. Panel E shows treatment with nontargeting radioconjugate. Each panel shows the tumor growth in individual animals. The significant therapeutic effect due to the combination is seen in panel D. Reproduced from Figure 2 of Mattes et al.[94]

Sharkey et al. extended this study to examine the effects of anti-CD20 immunotherapy using veltuzumab on either $^{90}$Y-veltuzumab RAIT or a pretargeting protocol involving a DNL construct, TF4, incorporating veltuzumab's specificity and a $^{90}$Y-labeled peptide[96]. Herein, both the agents targeted the same antigen. They also examined the effect of other naked antibodies, such as epratuzumab (anti-CD22) or milatuzumab (anti-CD74), and concluded that immunotherapy with veltuzumab given after RAIT offered the best improved outcome and that any predose of naked antibody should be minimized.

Further reiterating the importance of combination therapy in cancer treatments, Karacay et al. reported recently on a promising preclinical experiment combining $^{90}$Y-hPAM4 RAIT with hRS7-SN-38 conjugate (ADC) treatment in the Capan-1 model of pancreatic cancer[97]. In this, hPAM4 targeted a pancreas cancer antigen and hRS7 targeted EGP-1 (TROP-2) antigen on the tumor. The combination, with RAIT given first followed by a nontoxic dose of ADC given at a 25 mg/kg protein dose × 8 schedule, was better than either RAIT or ADC in terms of time-to-tumor progression and cures. When combining the MTD of RAIT (0.13 mCi/animal) with the ADC, 100% survival with 9/10 animals tumor-free was noted at >20 weeks, compared to 80% survival rate and 5/10 animals tumor-free with RAIT alone.

## CONCLUSIONS

Antibody-based targeted therapies of cancer offer opportunities for safer treatments. These have been generally more effective in hematological than in solid cancers. There are two approved products for RAIT of NHL. Recent advances from the authors' laboratories demonstrate the advantages of fractionated dosings in RAIT as well as combination therapies in the treatment of NHL, as well as pancreatic cancer. The superiority of pretargeted RAIT over using directly labeled MAbs, in terms of therapeutic indices, tumor regression, and PFS, has been established in preclinical models by a number of researchers and its contribution to clinical therapy is expected. New clinically validated technologies, such as DNL, can be also expected to impact the field of targeted therapy. In the area of ADCs, the clinical advance made with "T-DM1" for HER2-positive breast cancer and various other advances with different drugs and linker technologies are noteworthy. Especially interesting is the finding of the utility of noncleavable linker systems for increasing dose tolerability. The integration of emerging antibody-targeted therapies with other modalities should prove useful in the future management of cancer.

## ACKNOWLEDGMENTS

We thank our many collaborators for their contributions to the work summarized herein, particularly Drs. Robert M. Sharkey, Habibe Karacay, David V. Gold, Chien-Hsing Chang, Edmund A. Rossi, Thomas M. Cardillo, Bill McBride, Sung-Ju Moon, Gary L. Griffiths, Rhona Stein, and Hans J. Hansen.
